# The Surgical, Mechanical, and Medical Treatment of the Teeth, Including Dental Mechanics

**Published:** 1846-09

**Authors:** 


### Bibliographical Notices.

*The Surgical, Mechanical, and Medical Treatment of the Teeth: including Dental Mechanics.* Illustrated with one hundred and thirty-nine engravings. By JAMES ROBINSON, Surgeon Dentist to the Metropolitan Hospital; Lecturer on the Anatomy, Physiology and Pathology of the Teeth; Honorary Doctor of Dental Surgery of the Baltimore College of Dental Surgery, etc. etc. W. WEBSTER, London, LINDSAY & BLAKISTON, Philadelphia, 1846; pp. 320, 12mo.

WE have received from the author, a copy of the above named work, and have perused it with no little satisfaction. It is printed on very superior paper, and the mechanical execution, in every respect, is beautiful. The engravings are well gotten up, and contribute very greatly to enhance its value. It is well written, and embraces almost every subject coming properly within the province of dental surgery. The author is, evidently, not only a well-read dentist, but he is an experienced practitioner—a man of enlightened judgment—one whose opinion is entitled to consideration. There is also manifested in the work before us, a spirit of liberality, which is too seldom met with among the members of the dental profession.

The work being intended for the general as well as the professional reader, the author has, so far as practicable, avoided the use of technicalities, and minute details. In this, he has doubtless acted wisely, for while it will be more likely to be read by the former, it will prove a valuable manual to the latter.

The work is divided into two parts. The first, treats of the surgical, mechanical, and medical treatment of the teeth. In the first chapter of this part, the author glances very briefly at the history of dental surgery. He then commences with first dentition and the diseases which accompany it, the progress of the teeth of replacement, shedding of the temporary, &c. &c.

Chapters third, fourth, fifth, and sixth are devoted to "a comparative view of the teeth of man and animals," "the causes of irregularity of the teeth," mechanical means employed to remedy the same, as well as the means for its prevention; also, on new instruments for expanding the jaw, and on the causes and treatment of immobility of the lower maxilla.

In chapters seventh, eighth, ninth, tenth and eleventh, the author treats of the indications of the color of the teeth, the influence of the state of health upon the formation of these organs, their indications of age; the causes of dental caries, as well as those of tooth-ache and its treatment; the manner of filling teeth, materials proper to be employed in this operation; alveolar abscess; filing; salivary calculus and the manner of its removal.

Before we proceed farther, we would state, that in treating on the materials for filling teeth, Dr. Robinson gives gold a decided preference over every other article, and we are gratified to find that he condemns the use of that worst of all articles, amalgam, for this purpose.

Chapter twelfth is devoted to a description of the instruments employed for the extraction of teeth, and the manner of performing this operation. As a general rule he prefers the forceps, but in some cases he recommends the key-instrument. He seems to be somewhat incredulous with regard to the veracity of those who assert that they use no other instrument than the forceps. "Like any other instrument," he says, "it is adopted to particular cases only," and that "many young practitioners, fortified by the example of some of their elder professional brethren, assert that all their extractions are performed by that instrument. With their statistics of crushing, they do not furnish us, although these might help to set the question at issue between them and us, in a strong light."

Now, with regard to the superiority of properly constructed forceps, *in all cases*, over the key-instrument, we entertain no such doubts, although we are not much given to speaking of our own skill in any operation, we should have no objection to compare statistics with any one who uses the key-instrument, in this operation. We could also name many others, who would be glad of an opportunity of doing so. Indeed, so far from being correct in this country, is the implied insinuation of the author, that teeth are here daily extracted with forceps after having been broken with the key.—With properly constructed forceps, and the necessary care in their application, the crown of a tooth need not be crushed in one case in five hundred, and when an accident of this sort does occur, the tooth may always, by a second application, be removed. When we were in the habit of using the key-instrument, and we then had one as well adapted to the operation as any we have ever seen, we had the vanity to believe we could use it as expertly and successfully as most practitioners, but since we have laid it aside, and we have not owned one for more than twelve years, we have no hesitation in saying that we extract teeth with much greater ease, both to ourself and patient. Thus, our opportunity for testing the relative merits of the two instruments, has been ample, and candor compels us to give an unqualified preference to the forceps in *all cases*.

But to proceed: In chapter thirteenth and fourteenth, which are the last in part first of the work, the author notices the nervous affections of the face, which are often mistaken for tic doloureux; the importance of consulting medical practitioners; the value and importance of the teeth, means for preserving them; artificial teeth, and pivoted teeth.

Part second, treats of mechanical dentistry, in its various details, including the instruments and appliances required in this department of the art; surgical treatment of the mouth, previously to application of dental substitutes; the manner of separating silver, gold, and platina from other bodies; the importance of dentonomy and dental education.

Thus far, we have only noticed the general scope of the work under consideration, without examining the peculiar views of the author, which our limits, at present, will not permit us to do, and as most of our readers will have an opportunity of procuring it before the publication of the December number of the Journal, we must content ourself with the few general remarks which we have now made in relation to it. It may be had of Messrs. Lindsay & Blakiston, Philadelphia, the enterprising American publishers, and, we presume, at most of the bookstores in the country.—*Balt. Ed.*

